# The Antinociceptive Responses of MTDZ to Paclitaxel−Induced Peripheral Neuropathy and Acute Nociception in Mice: Behavioral, Pharmacological, and Biochemical Approaches

**DOI:** 10.3390/ph16091217

**Published:** 2023-08-29

**Authors:** Ketlyn P. da Motta, Carolina C. Martins, Vanessa M. Macedo, Beatriz F. dos Santos, Nelson Luís De C. Domingues, Cristiane Luchese, Ethel A. Wilhelm

**Affiliations:** 1 Biochemical Pharmacology Research Laboratory, LaFarBio, CCQFA, Federal University of Pelotas, UFPel, P.O. Box 354, Pelotas 96010-900, RS, Brazil; mottaketlyn@yahoo.com.br (K.P.d.M.); carol_cristovao@hotmail.com (C.C.M.); vnsmacedo@gmail.com (V.M.M.); 2 Organic Catalysis and Biocatalysis Laboratory, LACOB, Federal University of Grande Dourados, UFGD, P.O. Box 533, Dourados 79804-970, MS, Brazil; beatriz_biafuzinato@hotmail.com (B.F.d.S.); nelsondomingues@ufgd.edu.br (N.L.D.C.D.)

**Keywords:** paclitaxel, peripheral neuropathy, sulfur compound, nociception

## Abstract

The efficacy of 5-((4-methoxyphenyl)thio)benzo[c][1,2,5] thiodiazole (MTDZ) in mitigating paclitaxel (PTX)-induced peripheral neuropathy was investigated in male and female Swiss mice. The study examined the effects of MTDZ on various pathways, including transient receptor potential cation channel subfamily V member 1 (TRPV1), glutamatergic, nitrergic, guanylate cyclase (cGMP), serotonergic, and opioidergic. Mice received intraperitoneal PTX (2 mg/kg) or vehicle on days 1, 2, and 3, followed by oral MTDZ (1 mg/kg) or vehicle from days 3 to 14. Mechanical and thermal sensitivities were assessed using Von Frey and hot plate tests on days 8, 11, and 14. The open field test evaluated locomotion and exploration on day 12. On day 15, nitrite and nitrate (NOx) levels and Ca^2+^−ATPase activity in the cerebral cortex and spinal cord were measured after euthanizing the animals. MTDZ administration reversed the heightened mechanical and thermal sensitivities induced by PTX in male and female mice without affecting locomotion or exploration. MTDZ also modulated multiple pathways, including glutamatergic, NO/L−arginine/cGMP, serotonergic (5−HT_1A/1B_), opioid, and TRPV1 pathways. Additionally, MTDZ reduced NOx levels and modulated Ca^2+^−ATPase activity. In conclusion, MTDZ effectively alleviated PTX−induced peripheral neuropathy and demonstrated multi-targeted modulation of pain-related pathways. Its ability to modulate multiple pathways, reduce NOx levels, and modulate Ca^2+^−ATPase activity makes it a potential pharmacological candidate for peripheral neuropathy, acute nociceptive, and inflammatory conditions. Further research is needed to explore its therapeutic potential in these areas.

## 1. Introduction

Organosulfur compounds, which exhibit a wide range of pharmacological activities [1,2], are a class of drugs that constitute the majority of commonly used anti-inflammatories. Their therapeutic effects can be attributed to the abundance of sulfur in the body, which aids in several biological actions [1,3]. Among the organosulfur compounds, notable examples include meloxicam, acetylcysteine, nimesulide, and piroxicam [3]. Considering the pharmacological potential inherent in sulfur-containing drugs for the pharmaceutical industry, the exploration of organosulfur compounds exhibiting substantial anti-inflammatory and analgesic capacities holds promise for advancing pain management strategies [4,5].

**Scheme 1:**
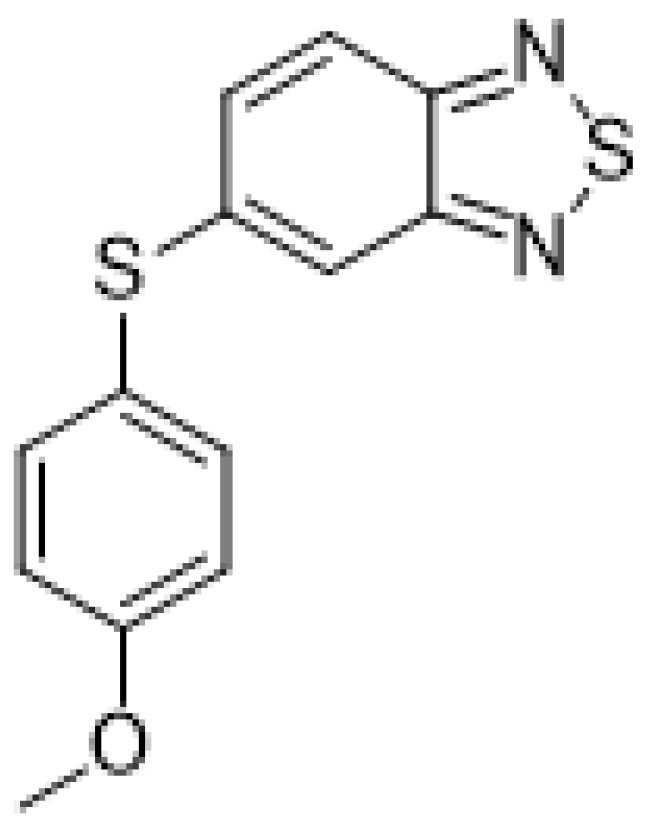
Structural design of 5-((4 methoxyphenyl)thio)benzo[c][1,2,5] thiodiazole (MTDZ).

In this context, 5-((4-methoxyphenyl)thio)benzo[c][1,2,5] thiodiazole (MTDZ) (Scheme 1), a synthetic compound structurally designed as a thiodiazole derivative containing a sulfur group, has shown promising pharmacological properties [6,7]. MTDZ has already shown pharmacological activities, including the potential to modulate the enzyme acetylcholinestare (AChE) in vitro and ex vivo [7]. Recently, the therapeutic potential of MTDZ against oxaliplatin-induced peripheral neuropathy has been observed [6]. MTDZ also exhibited analgesic potential at the central nervous system level, similar to or even better than morphine, in the acute hot plate test [6]. A previous study conducted by our research group [6] showed the potential of MTDZ to modulate alterations in the ionic gradient in the central nervous system through the action on ATPase enzymes, which is an intrinsic mechanism of neuropathy. Moreover, there are unknown pharmacological mechanisms of MTDZ that may contribute to its antinociceptive action occurring at both subchronic and acute levels. 

It is worth noting that some neurochemical events related to the activation of pain pathways are repeated in both acute and neuropathic pain, as suggested by Canta et al. [8]. This observation may explain the possible pharmacotherapeutic actions of the MTDZ compound under both painful conditions. In this context, Paclitaxel−induced peripheral neuropathy (PINP) is a debilitating oncological condition [9,10]. PTX causes the activation of inflammatory cascades and damage to the axonal electrochemical gradient, which play an essential role in the pathology of PTX−induced nerve damage and peripheral neuropathy [11,12]. Notably, MTDZ appears to be a relevant therapeutic strategy to investigate in relation to PINP. Moreover, understanding the acute antinociceptive potential of MTDZ can help in understanding the mechanistic ways in which MTDZ can act as a therapeutic agent.

In this study, our goal was to evaluate the therapeutic potential of MTDZ in both subchronic and acute pain. Firstly, we investigated the antinociceptive effects of MTDZ on male and female mice with PTX-induced peripheral neuropathy, analyzing the levels of nitrite and nitrate (NOx) as well as Ca^2+^−ATPase activity. Subsequently, we assessed the potential of MTDZ in acute nociception and investigated its pharmacological actions via the N−Methyl−D−Aspartate (NMDA)/nitric oxide (NO)/cyclic guanosine monophosphate (cGMP) pathway, the serotonergic pathway, the transient receptor potential cation channel subfamily V member 1 (TRPV1), and opioids.

## 2. Results

### 2.1. Evaluation of the Nociceptive Potential of MTDZ against PTX−Induced Peripheral Neuropathy—Mechanical Sensitivity

**Figure 1:**
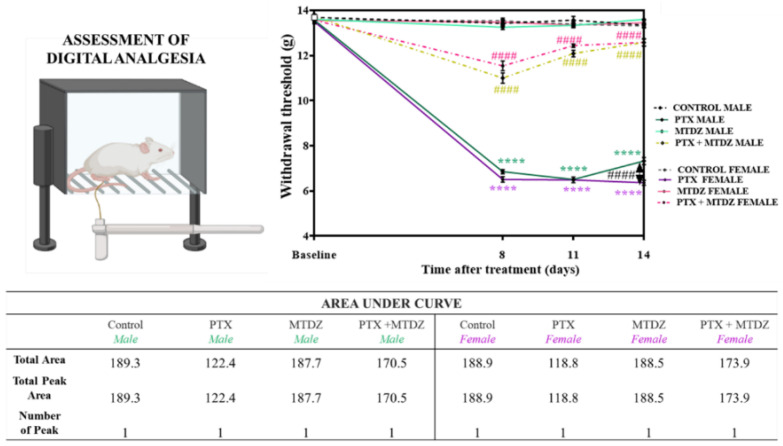
The impact of 5-((4-methoxyphenyl)thio)benzo[c][1,2,5] thiodiazole (MTDZ) and paclitaxel (PTX) on paw withdrawal threshold for assessment of mechanical sensitivity was evaluated on days 8, 11, and 14 of the experimental protocol in male and female mice. Each column within the graph represents the mean ± standard error of the mean (SEM) value of 7 mice in each respective group. (****) *p* < 0.0001 denotes significance levels when compared with the control group; (####) *p* < 0.0001 denotes significance levels when compared with the PTX group. Two-way ANOVA followed by the Tukey’s test was used.

Figure 1 illustrates the assessment of PTX−induced mechanical sensitivity and the therapeutic potential of MTDZ on days 8, 11, and 14 of the experimental protocol. As a preliminary baseline assessment of mechanical sensitivity, it was observed that none of the animals were sensitive before induction. On day 8, there was an increase in mechanical sensitivity in both male (49%) and female mice (53%). On day 11 of the experimental protocol, mechanical hypernociception generated by PTX was also observed in males and females (52%). On day 14, PTX caused mechanical hyperalgesia, with an increase of about 45% in males and 53% in females. However, females that received PTX showed an exacerbated response to mechanical sensitivity compared to males (15%). MTDZ reversed the mechanical sensitivity induced by PTX on all observation days in male and female mice. To aid in the interpretation and understanding of the statistical data, we calculated the area under the curve for each experimental group. 

### 2.2. Evaluation of the Nociceptive Potential of MTDZ against PTX−Induced Peripheral Neuropathy—Thermal Sensitivity 

**Figure 2:**
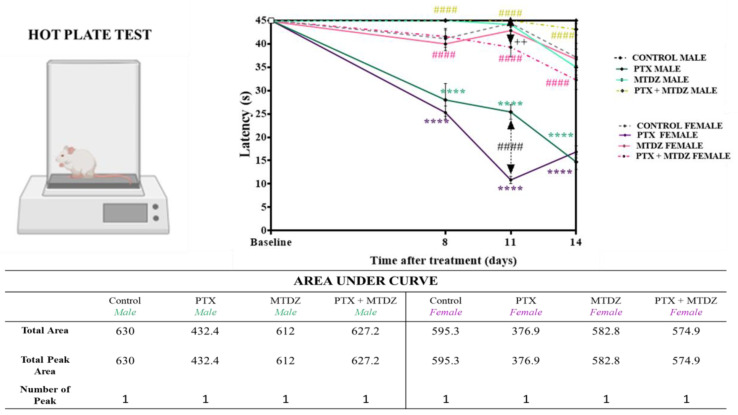
The impact of 5-((4 methoxyphenyl)thio)benzo[c][1,2,5] thiodiazole (MTDZ) and paclitaxel (PTX) was evaluated on temperature sensitivity in the hot plate test on days 8, 11, and 14 of the experimental protocol in male and female mice. Each column within the graph represents the mean ± standard error of the mean (SEM) value of 7 mice in each respective group. (****) *p* < 0.0001 denotes significance levels when compared with the control group; (^####^) *p* < 0.0001 denotes significance levels when compared with the PTX group and (^++^) *p* < 0.01 denotes significance levels when compared with the PTX+ MTDZ male. The green (males) and purple (females) colors indicate significance levels when the comparison indicated the difference between the same sex. The black color on days 11 and 14 indicates the difference in the nociceptive response between males and females. Two-way ANOVA followed by the Tukey’s test was used.

The effect of MTDZ as a therapeutic agent on PTX−induced thermal nociception is depicted in Figure 2. As a preliminary baseline assessment of thermal sensitivity, none of the animals were found to be sensitive before induction. On day 8 of the experimental protocol, there was a dramatic increase in thermal sensitivity in both male (38%) and female (38%) mice after PTX induction. On day 11, PTX−induced thermal sensitivity was observed in males (43%) and females (75%), with females showing an exacerbated response compared to males (32%) until day 14 of the experiment. However, on day 14, the thermal sensitivity induced by PTX in males (67%) and females (54%) remained increased compared to control animals.

MTDZ reversed the thermal sensitivity caused by PTX in male and female mice on all observation days. MTDZ was able to reverse thermal sensitivity on day 8 in males (61%) and females (64%); on day 11 in males (77%) and females (261%); and on day 14 in males (193%) and females (91%). To aid in interpreting and understanding the statistical data, we calculated the area under the curve for each experimental group.

### 2.3. Open Field Test

**Figure 3:**
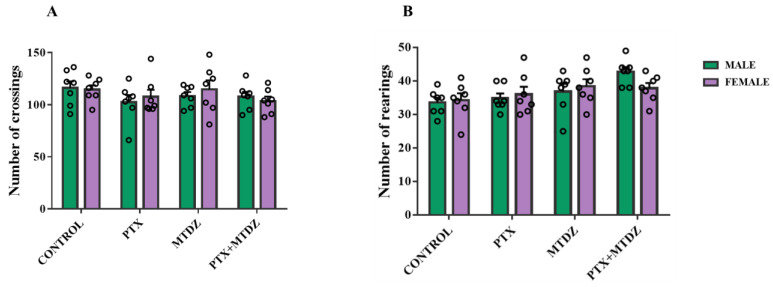
The impact of 5-((4-methoxyphenyl)thio)benzo[c][1,2,5] thiodiazole (MTDZ) and paclitaxel (PTX) on the open field test. Crossings (**A**) and rearings (**B**) in locomotion and exploration in male and female mice. Each column within the graph represents the mean ± standard error of the mean (SEM) value of 7 mice in each respective group. Two-way ANOVA followed by the Tukey’s test was used.

Figure 3 displays the counts of crossings and rearings observed during the open field test. Of note, there were no statistical distinctions in terms of locomotion (crossing) and exploratory actions (rearing) observed between all groups.

### 2.4. Biochemical Assays

#### 2.4.1. Ca^2+^−ATPase Activity

**Figure 4:**
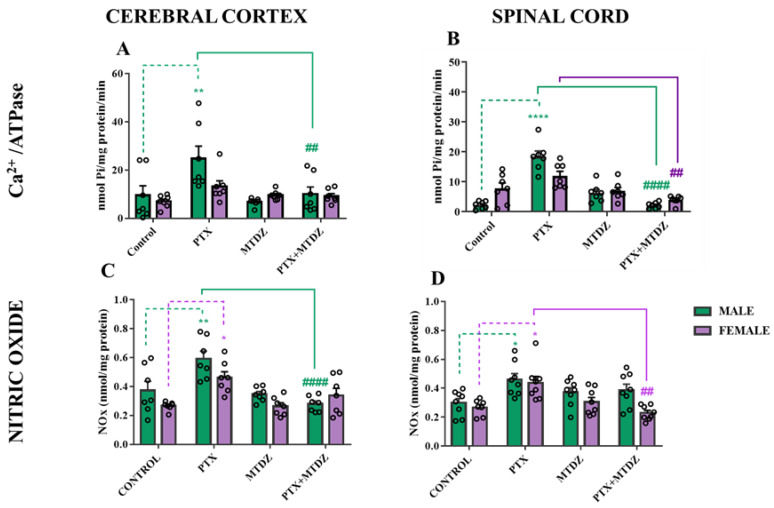
The impact of 5-((4-methoxyphenyl)thio)benzo[c][1,2,5] thiodiazole (MTDZ) and paclitaxel (PTX) was evaluated on Ca^2+^−ATPase activity. Enzyme activity in the cerebral cortex (**A**) and spinal cord (**B**) along with nitric oxide levels in the cerebral cortex (**C**) and spinal cord (**D**) of male and female mice. Each column within the graph represents the mean ± standard error of the mean (SEM) value of 7 mice in each respective group. (*) *p* < 0.05; (**) *p* < 0.01 and (****) *p* < 0.0001 denote significance levels when compared with the control group; (^##^) *p* < 0.01 and (^####^) *p* < 0.0001 denote significance levels when compared with the PTX group. Two-way ANOVA followed by Tukey’s test was used.

As shown in Figure 4, the PTX exposure increased the Ca^2+^−ATPase activity in the cerebral cortex (159%) (Figure 4A) and in the spinal cord (703%) (Figure 4B) of male mice. The MTDZ treatment normalized the enzyme activity after PTX exposure in male mice. A decrease in the activity of the Ca^2+^−ATPase enzyme was also observed in the PTX + MTDZ group compared to the PTX group of female mice. However, there were no alterations observed in the activity of the Ca^2+^−ATPase enzyme in the cerebral cortex and spinal cord of females.

#### 2.4.2. Nitric Oxide Levels

To investigate the mechanistic effects of MTDZ on the nitrergic pathway, we evaluated the levels of NOx in the cerebral cortex and spinal cord of mice exposed to PTX (Figure 4C,D). Our results showed that exposure to PTX increased nitric oxide levels in the cerebral cortex of both male (54%) and female (64%) mice, and in the spinal cord of both male (59%) and female (73%) mice. However, treatment with MTDZ reversed the increase in NOx in the cerebral cortex of male mice and in the spinal cord of female mice exposed to PTX.

### 2.5. Evaluation of the Modulating Potential of Transient Receptor Potential Vanilloid 1 (TRPV1), and Glutamatergic, Nitrergic, Serotonergic, and Opioidergic Pathways by MTDZ 

#### 2.5.1. Glutamatergic Pathway

**Figure 5:**
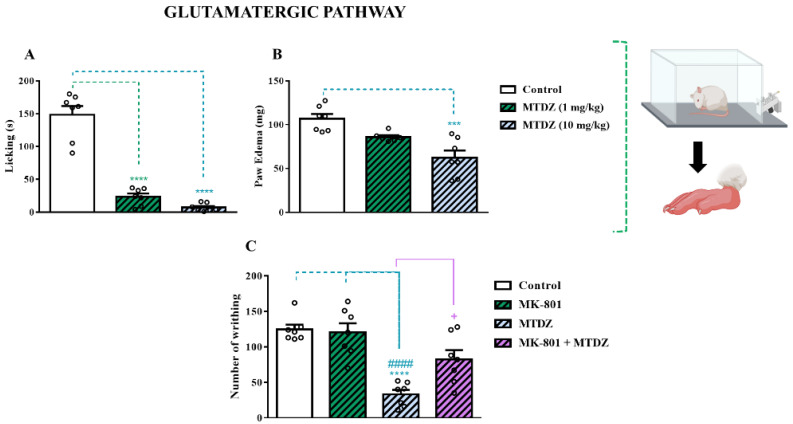
Antinociceptive effect of 5-((4 methoxyphenyl)thio)benzo[c][1,2,5] thiodiazole (MTDZ) (at 1 and 10 mg/kg, p.o.) on the licking behavior induced by glutamate in mice (**A**). (****) *p* < 0.0001 denotes significance levels when compared with the control group. Anti-inflammatory effect of MTDZ (at 1 and 10 mg/kg, p.o.) on the paw edema induced by glutamate in mice (**B**). (***) *p* < 0.001 denotes significance levels when compared with the control group. Effect of MK−801 (0.02 mg/kg, i.p.) on the antinociceptive action of MTDZ (10 mg/kg, p.o.) on the writhing behavior induced by acetic acid in mice (**C**). (****) *p* < 0.0001 denotes significance levels when compared with the control group; (^####^) *p* < 0.0001 denotes significance levels when compared with the MK−801 group and (^+^) *p* < 0.05 denotes significance levels when compared with the MTDZ group. Each column within the graph represents the mean ± standard error of the mean (SEM) value of 7 mice in each respective group. One-way ANOVA followed by the Tukey’s test was used.

The glutamatergic pathway is primarily responsible for synaptic excitability within the nervous system, and this cascade of events is closely linked to the signaling of the painful process. First, a curve dose-response of MTDZ in the glutamate test was performed to evaluate its antinociceptive activity. As shown in Figure 5A, MTDZ (at 1 and 10 mg/kg) reduced the nociceptive behavior induced by glutamate by 84% and 95%, respectively. Regarding the formation of edema by glutamate, it was observed that the MTDZ compound (at 10 mg/kg) reduced the edema formation by 41%.

Second, the involvement of the NMDA receptor in the acute antinociceptive action of MTDZ was evaluated by administering MK−801, a non-competitive NMDA receptor antagonist (Figure 5C). The results obtained showed that MK−801 administration blocked the antinociceptive activity of MTDZ in the acetic acid test. It is also worth noting that, as shown in Figure 5C, no significant differences were observed between the MK−801 and MK−801+MTDZ groups, indicating that the antinociceptive action of MTDZ is entirely due to the mediation of NMDA receptors.

#### 2.5.2. Nitrergic Pathways and Guanylate Cyclase 

**Figure 6:**
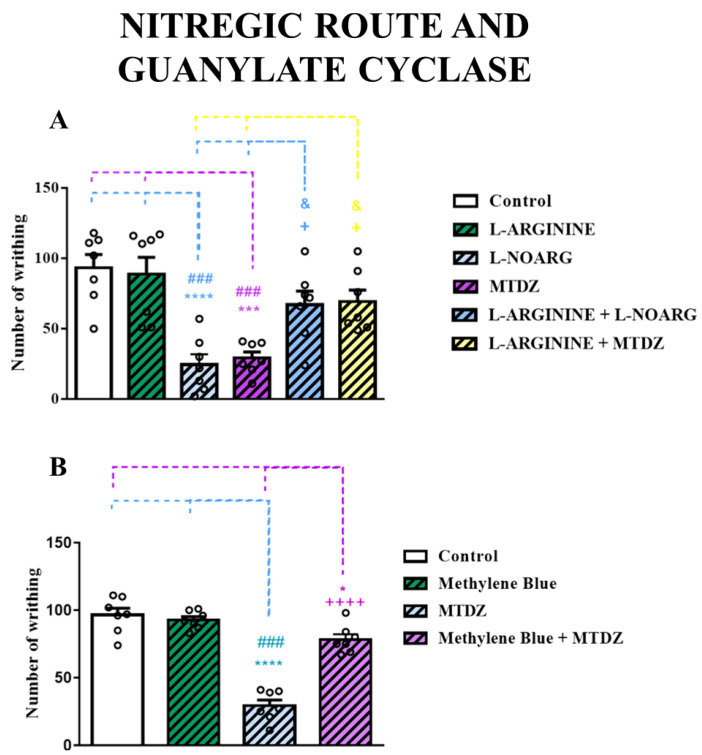
Effect of L−arginine (600 mg/kg, i.p.) on the antinociceptive action of 5-((4 methoxyphenyl)thio)benzo[c][1,2,5] thiodiazole (MTDZ) (10 mg/kg, p.o.) and L−NOARG (75 mg/kg, i.p.) on the writhing behavior induced by acetic acid in mice (**A**). Effect of methylene blue (10 mg/kg, i.p.) on the antinociceptive action of MTDZ (10 mg/kg, p.o.) on the writhing behavior induced by acetic acid in mice (**B**). (*) *p* < 0.05, (***) *p* < 0.001 and (****) *p* < 0.0001 denote significance levels when compared with the control group; (^###^) *p* < 0.001 denotes significance levels when compared with the L−arginine or methylene blue group; (^+^) *p* < 0.05 and (^++++^) *p* < 0.0001 denotes significance levels when compared MTDZ group and (^&^) *p* < 0.05 denotes significance levels when compared with L−NOARG group. Each column within the graph represents the mean ± standard error of the mean (SEM) value of 7 mice in each respective group. One-way ANOVA followed by the Tukey’s test was used.

The results in Figure 6B show that the animals treated with L−arginine (a precursor of nitric oxide formation) exhibited nociceptive behavior similar to the control group. However, both L−NOARG and MTDZ per se reduced the number of writhing compared with the control group. Moreover, the antinociceptive potential of L−NOARG or MTDZ was observed in animals pre-treated with L−arginine. It is also noteworthy that the effect of MTDZ is entirely due to the mediation on L−arginine, as shown in Figure 6A.

Methylene blue, a non-specific inhibitor of NO/guanylyl cyclase, can help inhibit NO levels above baseline. The group of animals that received methylene blue and control were statistically equal in acetic acid-induced nociception (Figure 6B). MTDZ reversed the nociception generated by acetic acid. On the other hand, when animals were treated with both methylene blue and MTDZ, an increase in nociception was observed. It should also be noted that the antinociceptive effect of MTDZ is entirely due to the mediation on guanylyl cyclase, as there were no differences between methylene blue and the methylene blue + MTDZ group.

#### 2.5.3. Serotonergic Pathway 

**Figure 7:**
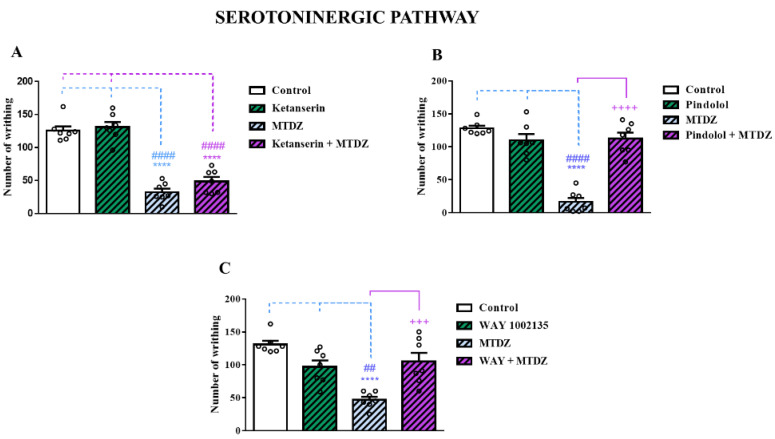
Effect of ketanserin (0.3 mg/kg, i.p.) on the antinociceptive action of 5-((4 methoxyphenyl)thio)benzo[c][1,2,5] thiodiazole (MTDZ) (10 mg/kg, p.o.) on the writhing behavior induced by acetic acid in mice (**A**). Effect of pindolol (1 mg/kg, i.p.) on the antinociceptive action of MTDZ (10 mg/kg, p.o.) on the writhing behavior induced by acetic acid in mice (**B**). Effect of WAY100635 (0.7 mg/kg, i.p) on the antinociceptive action of MTDZ (10 mg/kg, p.o.) on the writhing behavior induced by acetic acid in mice (**C**). (****) *p* < 0.0001 denotes significance levels when compared with the control group; (^##^) *p* < 0.01, (^####^) *p* < 0.0001 denotes significance levels when compared with the antagonist group (ketanserin, pindolol, and WAY100635); and (^+++^) *p* < 0.001, (^++++^) *p* < 0.0001 denotes significance levels when compared with the MTDZ group. Each column within the graph represents the mean ± standard error of the mean (SEM) value of 7 mice in each respective group. One-way ANOVA followed by the Tukey’s test was used.

The modulatory effect of MTDZ on the serotonergic pathway was demonstrated in Figure 7, using specific and non-specific antagonists. Figure 7A shows that pretreatment with ketanserin (a selective 5-HT_2A/2C_ receptor antagonist) did not affect the antinociceptive potential of MTDZ in the acetic acid test. However, pretreatment with pindolol (an antagonist of beta-adrenergic receptors B1 and B2, as well as 5−HT_1A/1B_ receptors) blocked the antinociceptive effect of MTDZ, as shown in Figure 7B. In addition, Figure 7C demonstrates that pretreatment with WAY100135 (a selective antagonist at the 5−HT_1A_ receptor) abolished the antinociceptive effect of MTDZ in the acetic acid test. 

It is also noteworthy that the antinociceptive effect of MTDZ is entirely due to the mediation on 5HT_1A/1B_ receptors (Figure 7B,C). This action was verified since there were no differences between the pindolol group and the pindolol + MTDZ group (Figure 7B) and between WAY1002135 and WAY1002135 + MTDZ (Figure 7C).

#### 2.5.4. Opioidergic and Transient Vanilloid Receptor Type 1 Pathway

**Figure 8:**
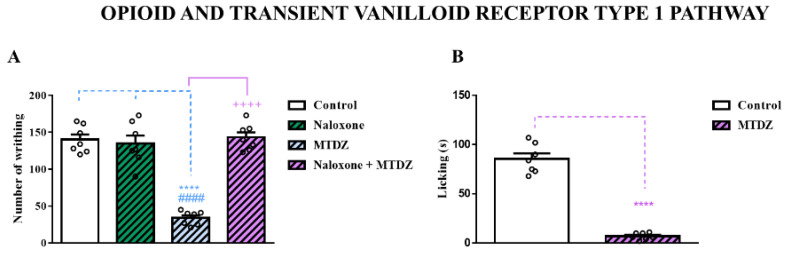
Effect of naloxone (5 mg/kg, i.p.) on the antinociceptive action of 5-((4 methoxyphenyl)thio)benzo[c][1,2,5] thiodiazole (MTDZ) (10 mg/kg, p.o.) on the writhing behavior induced by acetic acid in mice (**A**). (****) *p* < 0.0001 denotes significance levels when compared with the control group; (^####^) *p* < 0.0001 denotes significance levels when compared with the naloxone group and (^++++^) *p* < 0.0001 denotes significance levels when compared with the naloxone + MTDZ group. Effect of capsaicin (1.6 μg/paw) on the antinociceptive action of MTDZ (10 mg/kg, p.o.) (**B**). (****) *p* < 0.0001 denotes significance levels when compared with the control group. Each column within the graph represents the mean ± standard error of the mean (SEM) value of 7 mice in each respective group. One-way ANOVA followed by the Tukey’s test.

The opioidergic pathway and TRPV1 are target mechanisms for therapeutic modulation of analgesic drugs. Our results demonstrated that pretreatment with naloxone (a non-specific antagonist of opioid receptors, more selective for µ and κ receptors) blocked the antinociceptive effect of the MTDZ compound against the nociception induced by acetic acid (Figure 8A). As shown in Figure 8B, the modulatory potential of TRPV1 by MTDZ after administration of capsaicin (an agonist of TRPV1 associated with the nociceptive process) was evidenced. The MTDZ compound was able to reverse the agonist effect of capsaicin on TRPV1.

## 3. Discussion

The present study provides novel evidence supporting the potential of MTDZ as a promising therapeutic strategy for the treatment of PTX-induced peripheral neuropathy. Our findings also shed light on the underlying mechanisms through which MTDZ exerts its significant antinociceptive effects in acute and chronic pain. Interestingly, our results demonstrate that PTX induces both mechanical and thermal sensitivities in male and female mice, with females displaying exacerbated nociceptive responses on days 14 and 11 of the experimental protocol, respectively. Moreover, we observed an involvement of the Ca^2+^−ATPase enzyme and elevated NOx levels in the cerebral cortex and spinal cord, which likely contribute to the development of peripheral neuropathy. In contrast, MTDZ administration effectively reversed PTX−induced mechanical and thermal nociception, while normalizing Ca^2+^−ATPase activity and NOx levels in male and/or female mice.

Furthermore, our study elucidated that the acute antinociceptive properties of MTDZ primarily involve the modulation of NMDA/NO/cGMP pathways, serotonergic 5HT_1A/1B_ receptors, TRPV1, and opioid receptors. The detrimental impact of PTX on the central and peripheral nervous system results in alterations in neuronal sensitivity and excitability [13]. Sensory neurons, particularly sensory fibers, are profoundly affected by PTX, leading to amplified sensory responses to mechanical and thermal stimuli [12]. Consistent with these findings, our study demonstrated that PTX exposure elicited nociceptive responses to mechanical and thermal stimuli in both male and female mice on days 8, 11, and 14 of the experimental protocol. Notably, we observed an exacerbation of mechanical sensitivity in females on the 14th day, highlighting a sex-dependent discrepancy in nociceptive responses that had not been statistically analyzed in previous studies [14]. Additionally, our study, for the first time, reveals that female mice exposed to PTX exhibit heightened responsiveness to thermal stimuli compared to males, particularly on the 11th day of the experimental protocol.

The likelihood of developing neuropathic pain is recognized to be greater among female patients [14,15]. One factor that could contribute to this difference is the higher density of nerve fibers in women compared to men [4]. Interestingly, studies suggest that females may exhibit greater adaptability to pain compared to males, particularly in response to prolonged noxious stimuli such as sustained heat pain [4,16]. Our findings are consistent with this hypothesis, as females showed greater thermal sensitivity on day 11, which then returned to the threshold observed in males.

In contrast, MTDZ effectively reversed the mechanical and thermal sensitivities induced by PTX on all observed days in both male and female mice. Additionally, hormonal factors may play a role in the observed nociceptive responses, as females generally exhibit higher pain sensitivity [17]. Therefore, MTDZ represents an intriguing candidate for the treatment of chemotherapy-induced peripheral neuropathy, given its promising effects in a neuropathy model induced by oxaliplatin [6].

Upon confirming the establishment of neuropathic conditions in the animals, as evidenced by increased mechanical and thermal sensitivities, we observed biochemical changes in the cerebral cortex and spinal cord that could contribute to the understanding of PTX−induced neuropathy pathophysiology. Alterations in ATPase enzyme homeostasis often indicate impaired axonal function, leading to changes in excitability and nerve conduction [18]. Our study revealed a sex-specific increase in Ca^2+^−ATPase activity in the cerebral cortex and spinal cord of male mice exposed to PTX. Indeed, intracellular Ca^2+^ dysregulation has been associated with increased mitochondrial damage, leading to nerve fiber injury, neuropathic pain, thermal sensitivity, and axonal degeneration [9]. Importantly, it was previously unknown that these Ca^2+^−related changes induced by PTX were sex-specific. Our study is pioneering in reporting that the increase in Ca^2+^−ATPase activity is more closely associated with nociceptive sensitivity in male mice exposed to PTX compared to female mice. On the other hand, MTDZ was found to normalize Ca^2+^−ATPase activity when it was altered by PTX exposure. This novel finding regarding specific alterations in Ca^2+^−ATPase activity in male mice expands our understanding of potential key mechanisms that contribute more significantly to the development of pain pathology in males compared to females.

Hypotheses have suggested that PTX can interact with neuronal Ca^2+^ sensor 1 (NCS-1), resulting in a positive modulation of the inositol 1,4,5-triphosphate receptor (InsP3R) [19]. This interaction leads to changes in Ca^2+^ concentration, which, in turn, activate proteases that damage peripheral neurons and induce apoptosis [20]. Therefore, drugs that specifically modulate Ca^2+^ signaling and the activity of Ca^2+^−ATPase, such as MTDZ, hold promise as therapeutic alternatives for PTX−induced peripheral neuropathy.

In our study, we observed changes in NOx levels in the cerebral cortex and spinal cord of mice. PTX administration resulted in increased NOx levels in both male and female mice. The initiation of inflammatory processes, characterized by elevated pro-inflammatory cytokines, is associated with the upregulation of inducible nitric oxide synthase (iNOS), leading to increased NOx production and the activation of inflammatory and immunological responses. Unlike other chemotherapeutic agents that induce neuropathic pain, PTX exacerbates inflammatory conditions, contributing to the pathophysiology of peripheral neuropathy [9]. The involvement of the nitrergic pathway in PTX−induced neuropathic pain has been well documented, and modulation of this pathway holds promise for its treatment [21,22]. Interestingly, MTDZ administration reversed the elevated NOx levels in the cerebral cortex of male mice and in the spinal cord of female mice exposed to PTX. These findings highlight both tissue-specific and sex-specific modulation of MTDZ in response to the deleterious effects of PTX.

Overall, our study provides valuable insights into the complex interplay between PTX, Ca^2+^ signaling, and the nitrergic pathway in the development of peripheral neuropathy. MTDZ emerges as a potential therapeutic intervention, capable of modulating these pathways and mitigating the harmful effects of PTX on Ca^2+^ homeostasis and NOx production.

The promising potential of MTDZ in treating PTX−induced peripheral neuropathy and the need to gain a better understanding of the modulated pathways underlying its acute antinociceptive effects motivated us to conduct a more focused investigation. Specifically, we aimed to explore the neurospecific actions of MTDZ and elucidate the mechanistic pathways involved in its antinociceptive activity. We emphasize that the substances used as antagonists or agonists of the pathways studied here may indirectly exert action on receptors or secondary pathways. Of these drugs, pindolol is described for acting on beta-adrenergic receptors; MK−801 can act on nicotinic receptors; and naloxone can act on the NMDA receptor.

To this end, we initially assessed the modulation of the glutamatergic and nitrergic pathways, along with the evaluation of cGMP modulation. These pathways, particularly the NMDA receptor, NOx modulation (nitrergic pathway), and cGMP pathway, collectively constitute a pivotal mechanism for combating nociception (NMDA/NO/cGMP pathway) [23].

Indeed, the administration of the glutamate test typically induces an inflammatory response characterized by vasodilation and extravasation of exudate, leading to paw edema. In our study, MTDZ demonstrated the ability to attenuate the nociceptive behavior of paw licking induced by glutamate at a dose of 10 mg/kg. Moreover, at the same dose, MTDZ prevented the establishment of the inflammatory process by reducing paw edema caused by glutamate. Notably, the glutamate−induced nociception model involves the activation of non-NMDA ionotropic receptors, namely kainate and α-amino-3-hydroxy-5-methyl-4 isoxazolpropionate (AMPA) receptors [24]. Consequently, this result indirectly suggests the potential modulation of MTDZ on AMPA and kainate receptors, contributing to its antinociceptive effects.

Glutamate, acting through the NMDA receptor, stimulates NO synthase. Interestingly, we also observed that MTDZ was capable of reducing the nociception caused by L−arginine. L−arginine serves as a precursor for NOx formation, which is mediated by NO synthase. Furthermore, when L-arginine and MTDZ were combined, there was a decrease in nociception commonly induced by L−arginine. Similarly, the association of L−arginine with L−NOARG, a NO synthase inhibitor, further reduced nociception. These findings suggest that MTDZ exerts effects similar to those of L−NOARG, preventing the formation of NOx. Considering the NMDA/NO ratio, it can be inferred that MTDZ not only acts on the NMDA receptor but also decreases the inflammatory process by reducing NOx production.

To further elucidate the NMDA/NO/cGMP pathway, we investigated the nociception induced by acetic acid in the presence of methylene blue with MTDZ. Methylene blue is a soluble inhibitor of cGMP. cGMP is directly associated with NO signaling. Excessive NOx can activate soluble guanylyl cyclase (GCs), leading to the conversion of guanosine triphosphate (GTP) into cGMP and inorganic phosphate. Throughout this entire NMDA/NO/cGMP mechanism, pain processing is regulated. Our results indicated that MTDZ acts through the cGMP pathway, as its antinociceptive effects were interrupted when combined with methylene blue. These findings provide valuable insights into the mechanistic pathways modulated by MTDZ, highlighting its ability to interfere with glutamate receptors, reduce NOx formation, and impact cGMP signaling. The modulation of the NMDA/NO/cGMP pathway by MTDZ contributes to its antinociceptive properties, presenting it as a promising therapeutic option for the treatment of pain.

In regard to the serotonergic pathway, MTDZ was found to specifically target 5-HT_1A/1B_ receptors. Descending pain pathways often involve serotonin receptors, with neuropathic pain in particular affecting transmission through descending serotonergic pathways by altering 5−HT_1A/1B_ receptors [25]. Notably, the dorsal horn, where pain processing occurs, predominantly contains the 5−HT_1_ receptor subtype [25]. Selective modulation of the 5−HT_1A_ subtype may exert a significant inhibitory effect against mechanical hyperalgesia [26]. In the research conducted by Sagalajev et al. [27], an exploration was carried out into the mechanisms through which the central nucleus of the amygdala regulates mechanical hyperalgesia in the descending nociceptive pathway of rats. The study revealed the participation of 5−HT1A receptors in this process.

Additionally, MTDZ exhibited modulation of nociceptive responses through the opioid pathway and TRPV1, as evidenced in the capsaicin test. TRPV1 is found in the somatosensory system, primarily in the primary afferent neurons responsible for sensing pain stimuli. Activation of TRPV1 leads to a burning sensation by promoting nerve impulses that contribute to central stimulation in the pain pathway [28]. Consequently, TRPV1 represents a pharmacological target for pain treatment, including chronic pain, cancer pain, neuropathic pain, and musculoskeletal pain [11,28]. Drugs that act on TRPV1 can modulate the transcriptional regulation of the receptor, reducing the expression of pro-nociceptive molecules and peripheral sensitization [11]. Thus, the antinociceptive action of MTDZ through TRPV1 suggests its potential efficacy in alleviating the burning sensation caused by TRPV1 activation and its ability to regulate transcriptional modulation by reducing the expression of pro-nociceptive molecules.

Regarding the opioidergic pathway, a previous study demonstrated that MTDZ exhibited similar or even superior antinociceptive effects compared to morphine in an acute pain model. Given its potential to modulate the opioid pathway, it is hypothesized that MTDZ possesses strong analgesic activity, as it has previously shown superior effects to morphine [6]. In fact, the antinociceptive action of MTDZ was blocked by treatment with the opioid receptor antagonist naloxone. These findings suggest that the antinociceptive action of MTDZ, as previously demonstrated, may occur through modulation of µ and κ opioid receptors.

**Figure 9:**
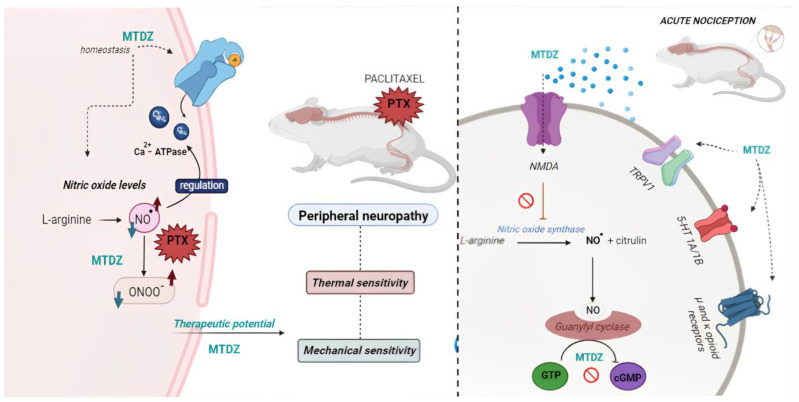
Graphic summary of the main results obtained in the present study of the effects of compound 5−((4 methoxyphenyl)thio)benzo[c][1,2,5] thiodiazole (MTDZ) in acute nociception and neuropathic pain. In paclitaxel (PTX) − induced peripheral neuropathy, MTDZ relieves mechanical and thermal sensitivities by reducing levels of nitrites and nitrates (including nitric oxide radical (NO^•^) and peroxynitrite (ONOO^•^)) that are elevated due to PTX administration (left side of image). Furthermore, MTDZ restores the activity of the Ca^2+^−ATPase enzyme, maintaining its homeostasis in males exposed to PTX. In acute nociception, MTDZ modulates acetic acid−induced nociceptive responses through regulatory actions on the N−Methyl−D−Aspartate (NMDA)/nitric oxide (NO)/cyclic guanosine monophosphate (cGMP) pathway, the V subfamily of transient receptor potential cation channel limb 1 (TRPV1), the 5HT_1A/1B_ receptors, as well as the µ and κ opioid receptors (right side of image).

Considering the collective results presented in this study, MTDZ emerges as a promising therapeutic compound for the treatment of acute and subchronic nociception (Figure 9). MTDZ effectively mitigated nociception induced by PTX−induced peripheral neuropathy, even when females exhibited heightened mechanical and thermal sensitivities compared to males. The therapeutic potential of MTDZ at the subchronic level is attributed to its ability to modulate the Ca^2+^−ATPase enzyme and NOx levels in the cerebral cortex and spinal cord, both of which are pathways altered by PTX chemotherapy. Moreover, in relation to acute nociception, MTDZ’s neurotherapeutic action appears to involve the NMDA/NO/cGMP pathways, serotonergic modulation of 5−HT_1A/1B_ receptors, TRPV1 activation, and the opioid receptor system.

## 4. Material and Methods

### 4.1. Animals

The study was conducted in two stages. In the first step, we investigated the neuropathy caused by PTX and evaluated the therapeutic potential of MTDZ using male and female Swiss mice (60 days old). The investigation of sex differences was crucial due to the known neurotoxic effects of PTX. In the second step of the study, we focused on investigating the pathways involved in the acute antinociceptive action of MTDZ. To prioritize animal welfare and minimize the number of animals used, this phase exclusively utilized male Swiss mice (60 days old).

All procedures followed the ethical guidelines set by the Committee on Ethics and Animal Experimentation of the Federal University of Pelotas, Brazil (CEUA/UFPel 12/2022). The animals were housed in standard cages placed in rooms maintained at a temperature of 22 ± 2 °C. They had ad libitum access to food and water and were subjected to a 12-h light/dark cycle, with lights on at 07:00 h. We made every possible effort to minimize both the number of animals used and any potential discomfort they may have experienced during the experiments.

Seven animals per group were used in all experiments, with a total of 265 animals being used. Of these, for the subchronic study, about 28 males and 28 females were used. The investigation stage of the mechanistic pathways of MTDZ involved the use of only male mice to reduce the use of animals as much as possible. The female gender in mice evaluated in this research was seen by our group as necessary data to be considered. This is because peripheral neuropathy is a clinical condition with a higher incidence in women [16].

### 4.2. Drugs

The PTX was purchased from Indústria Farmin e Comércio Ltd.a (Guarulhos, São Paulo, Brazil) in the commercial form of Evotaxel^®^. The PTX was prepared from a concentration of 6 mg/mL to a dose of 2 mg/kg through dilution with solution for injections. The MTDZ (Scheme 1) was synthesized and characterized in the Catalysis and Biocatalysis Laboratory at the Federal University of Grande Dourados as previously described by Santos et al. [7]. The MTDZ’s purity, which stood at 99%, was assessed through column chromatography using exclusively hexane. The ^1^H and ^13^C NMR spectra were captured in CDCl_3_ using a Bruker spectrometer, operating at frequencies of 300 MHz and 75 MHz, respectively. For experimentation, the MTDZ was dissolved in canola oil, while PTX was dissolved in a 5% glucose solution.

The other drugs (naloxone, capsaicin, methylene blue, L−arginine hydrochloryde (L−arginine) and ω−nitro−L−arginine (L−NOARG), ketanserin, pindolol, WAY100635, acetic acid P.A., monosodium glutamate (MSG), and MK−801) were obtained commercially from Sigma−Aldrich (St. Louis, MO, USA). Ketanserin, WAY100635, MK−801, L−arginine, L−NOARG, methylene blue, capsaicin, MSG, acetic acid, and naloxone were dissolved in isotonic saline solution; pindolol was dissolved in Tween 80 (10%).

### 4.3. Evaluation of the Nociceptive Potential of MTDZ against PTX−Induced Peripheral Neuropathy

Peripheral neuropathy resulting from PTX currently lacks an effective therapeutic approach to address the series of neurotoxic events induced by PTX. Therefore, the potential of MTDZ was explored, primarily in the context of PTX−induced neuropathy, considering its previously demonstrated therapeutic efficacy in another neuropathy model. The dosage of 1 mg/kg of MTDZ was selected based on its therapeutic effectiveness in treating peripheral neuropathy induced by oxaliplatin in mice [6].

### 4.4. Experimental Design

**Figure 10:**
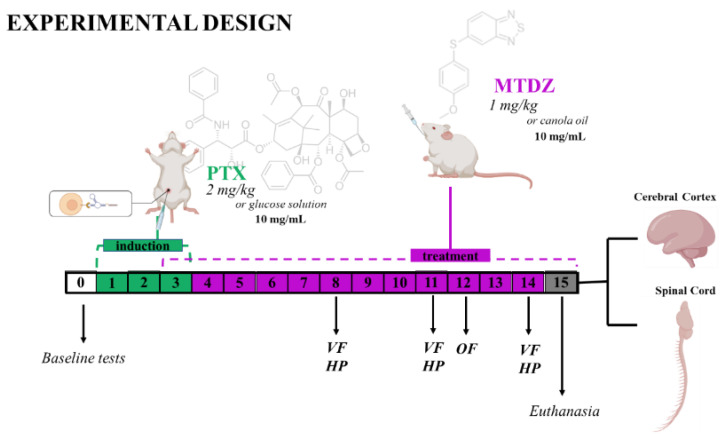
The experimental design. Male and female mice received paclitaxel (PTX) (2 mg/kg) or 5% glucose solution (10 mL/kg) intraperitoneally on days 1, 2, and 3 of the experimental protocol. Two experimental groups received treatment with 5-((4 methoxyphenyl)thio)benzo[c][1,2,5] thiodiazole (MTDZ) (1 mg/kg) for 12 days or its canola oil vehicle (10 mL/kg), per oral route. The animals were previously submitted to the paw withdrawal threshold stimulus in the von Frey (VF) and thermal stimulus in the hot plate test (HP). The VF and HP tests were verified on days 8, 11, and 14 of the experimental protocol. On day 12, the open field (OF) test was performed. On day 15, the animals were euthanized, and the cerebral cortex and spinal cord were dissected and frozen for further biochemical analysis.

Male and female Swiss mice were indiscriminately sorted into four distinct experimental cohorts: (1) Control, (2) PTX, (3) MTDZ, and (4) PTX + MTDZ. Throughout the initial three days, the control and MTDZ groups were administered a 5% glucose solution (10 mL/kg, via intraperitoneal injection (i.p.)), while the PTX and PTX + MTDZ groups received PTX (2 mg/kg, i.p.). Starting from day 3 until day 14 of the trial, the control and PTX groups were subjected to canola oil (10 mL/kg, administered orally via gavage (p.o.)), whereas the MTDZ and PTX + MTDZ groups were given a daily dose of MTDZ (1 mg/kg, p.o.). The assessment of mechanical and thermal sensitivities transpired on days 8, 11, and 14 of the trial. The open field (OF) test was conducted on day 12. At the culmination of the experimental regimen (day 15), the mice were humanely euthanized, and their cerebral cortex and spinal cord structures were harvested for subsequent analysis. The assessment days for mechanical and thermal sensitivities and the OF test were chosen based on previously established protocols involving chemotherapeutics [6,29] (Figure 10).

### 4.5. Nociceptive Behavior—Mechanical Sensitivity

The animals’ mechanical sensitivity was evaluated on days 0, 8, 11, and 14 of the experimental protocol following the methodology described by Alamri et al. [30]. Day 0 served as a baseline assessment to exclude animals with pre-existing sensitivity. For this behavioral test, the animals were placed in individual compartments within acrylic boxes, which were kept in a dark environment. The boxes were suspended using a metal apparatus supported by a grid. A digital analgesimeter was used to measure the paw withdrawal threshold of the animals. The polypropylene tip of the analgesimeter was applied perpendicularly to the middle of the plantar surface of the hind paw, exerting a progressively increasing pressure until the paw withdrawal response occurred. The pressure value at the moment of withdrawal was automatically recorded. To obtain individual results, each animal underwent six assessments of mechanical sensitivity, and an average value was calculated. The data were presented as paw withdrawal threshold (g).

### 4.6. Nociceptive Behavior—Thermal Sensitivity 

The animals’ thermal sensitivity was assessed on days 0, 8, 11, and 14 of the experimental protocol following the methodology outlined by Woolfe and MacDonald [31]. Day 0 served as a baseline assessment to exclude animals with pre-existing sensitivity. The animals were placed inside an acrylic box positioned on a metal platform that had been preheated to 52 ± 1 °C. The latency of nociceptive responses, such as paw licking, flapping, or jumping, was recorded as a measure of nociceptive behavior. To prevent tissue damage to the animals’ paws, a maximum time limit of 45 s was set for their stay on the platform. The results were reported in terms of latency (seconds).

### 4.7. Open Field (OF) Test

The open field (OF) test, as outlined in the procedure detailed by Walsh and Cummins [32], was employed to evaluate the mice’s overall locomotor and exploratory tendencies. The OF apparatus was fabricated from plywood and featured 30 cm high walls. The floor of the OF was divided into 9 quadrants, forming a layout of 3 rows by 3 quadrants each. On the twelfth day of the experimental regimen, the mice underwent the OF test. Placed at the center of the OF, each mouse was observed for a duration of 4 min to document their locomotor activity (number of quadrants traversed by all four paws) and exploratory conduct (frequency of hind limb rearings). A single trial was conducted for each mouse. The locomotion findings were presented in terms of the count of crossings, while exploration was quantified by the frequency of rearings.

### 4.8. Biochemical Assays

#### 4.8.1. Ca^2+^−ATPase Activity

Samples of the cerebral cortex and spinal cord were prepared in a 50 mM Tris-HCl buffer. Subsequently, the samples were centrifuged at 2500 rpm for 10 min to obtain the supernatant for further analysis. The Ca^2+^−ATPase activity was measured following the protocol described by Rohn et al. [33] and later adapted by Trevisan et al. [29]. The assay was carried out in an incubation system containing 30 mM Tris-HCl, 50 mM NaCl, 5 mM KCl, 0.4 mM CaCl_2_, 26 mM MgCl_2_, 3 mM ATP, and 50 μL of the prepared sample. To verify the enzymatic activity, all reaction salts, except for CaCl_2_, were included in a parallel incubation system. The activity of the enzyme was quantified in nmol of Pi/min/mg of protein.

#### 4.8.2. Nitric Oxide Levels

Cerebral cortex and spinal cord samples were prepared by immersing them in a mixture of ZnSO_4_ (200 mM) and acetonitrile (96%), after which they were subjected to centrifugation at 14,000 rpm for 30 min at 4 °C, in order to harness the supernatant. The Griess reaction [34] was employed to assess the presence of nitrite in the supernatant, which serves as an indicator of oxidation. The NOx content was gauged within a solution containing 2% vanadium chloride (dissolved in 5% HCl), 0.1% N-(1-naphthyl)ethylenediamine dihydrochloride, and 2% sulfanilamide (dissolved in 5% HCl). Following an hour of incubation at 37 °C, the color reaction was quantified through spectrophotometric measurements at 540 nm. The concentration of nitrite/nitrate within the supernatant was ascertained using a standard sodium nitrite curve and expressed as nmol NOx per gram of tissue.

### 4.9. Evaluation of the Modulating Potential Transient Receptor Potential Cation Channel Subfamily V Member 1 (TRPV1), and Glutamatergic, Nitrergic, Serotonergic, and Opioidergic Pathways by MTDZ

In this subsequent phase, prompted by the effects of MTDZ in treating PTX-induced neuropathy, we recognize the importance of investigating the underlying mechanistic pathways that contribute to MTDZ’s antinociceptive activity. Nociception was induced by acetic acid for substances that did not elicit nociceptive responses on their own, such as MK−801, ketanserin, pindolol, WAY100635, L−arginine, methylene blue, and naloxone. For the glutamate test, the inducer itself was MSG. To determine the appropriate dosage of MTDZ for studying its modulation pathways, two doses were initially tested in the glutamate test. The dose that yielded the most significant acute antinociceptive effects was selected. The selection of the route of administration, treatment duration, and dosage was based on previous studies by Silva et al. [35], Martins et al. [36], and Motta et al. [6].

#### 4.9.1. Acetic Acid Test

The acetic acid test was used as an inducer of nonspecific nociception to assess the involvement of different pathways, through antagonists, which may be contributing to the pharmacological potential of MTDZ. Abdominal writhing was induced by an injection of 450 µL of acetic acid (1.6%) [3]. After acetic acid injection (i.p.), mice were placed individually in separate boxes and abdominal constrictions were cumulatively counted over a period of 20 min. Results were expressed as the number of abdominal writhing. 

#### 4.9.2. Glutamatergic Pathway

MSG can cause morphological and electrophysiological changes in the nervous system, triggering an increase in pain sensitivity. As described by Beirith et al. [37], mice were pretreated with MTDZ (1 and 10 mg/kg, p.o.) or vehicle (canola oil, orally, 10 mL/kg) half an hour prior to MSG injection (20 μmol/paw, intraplantar) in the left hind paw, and saline (0.9%, 20 μL/paw) was injected into the right hind paw. After the injection of glutamate, the mice were individually observed for a duration of 15 min. The period during which the mice licked the paw into which MSG was injected was recorded using a stopwatch and regarded as indicative of nociceptive behavior. After the behavioral assessment, the mice were euthanized, and their paws were excised and weighed. The extent of paw edema was quantified by comparing the weight of the paw that received the MSG injection to the contralateral paw (treated with saline).

Additionally, an exploration into the participation of the N-methyl-d-aspartate (NMDA) glutamatergic receptor was undertaken. The role of the NMDA receptor in the analgesic effects of MTDZ was analyzed using MK−801 (0.02 mg/kg, intraperitoneal), a non-competitive NMDA receptor antagonist. A quarter of an hour following the administration of MK−801 or the vehicle (10 mL/kg, intraperitoneal), the animals were given MTDZ (10 mg/kg, orally). After 30 min, they underwent the acetic acid test as described earlier.

#### 4.9.3. Nitregic Route and Guanylate Cyclase Pathway

To verify the involvement of the nitrergic pathway in the antinociceptive effect of MTDZ, mice were pretreated with L−arginine, a precursor of NO (600 mg/kg, i.p.) or vehicle (10 mL/kg, i.p.), and 15 min later received MTDZ (10 mg/kg, kg, p.o.), ω−nitro−L−arginine (L−NOARG) (75 mg/kg, i.p., a NO synthase inhibitor), or the respective vehicle (10 mL/kg, i.p.). After 30 min, the mice received an injection of acetic acid (as described above).

Another set of animals received methylene blue (10 mg/kg, i.p.), administered 15 min before treatment with MTDZ (10 mg/kg, i.p.) [36]. After 30 min of treatment with MTDZ, the animals were injected with acetic acid as described above to investigate their nociception. 

#### 4.9.4. Serotonergic Pathway 

The possible contribution of the serotonergic system to the antinociceptive action of MTDZ was investigated using ketanserin (0.3 mg/kg, i.p., a selective antagonist of 5−HT_2A/2C_ receptor), pindolol (1 mg/kg, i.p., a nonselective antagonist of 5−HT_1A/1B_ receptors), and WAY100635 (0.7 mg/kg, i.p., a selective antagonist of 5−HT_1A_ receptor). Fifteen minutes after the antagonist or vehicle administration (10 mL/kg of body weight, i.p.), animals received MTDZ (10 mg/kg, p.o.) and after 30 min, acetic acid was injected (as described above). 

#### 4.9.5. Opioidergic and Transient Vanilloid Receptor Type 1 Pathway

Role of the opioid pathway and the TRPV1 receptor in pain signaling pathways is well-established. Therefore, we investigated the potential involvement of the opioid system in the antinociceptive effect of MTDZ using the acetic acid test, as described previously. In this experiment, the animals were pretreated with naloxone (5 mg/kg, i.p.) or saline (0.9% NaCl) 15 min before receiving either the vehicle (canola oil, p.o., 10 mL/kg) or MTDZ (10 mg/kg, p.o.).

To explore the mechanistic pathway of MTDZ’s antinociceptive action via the TRPV1 receptor, the animals were administered MTDZ (10 mg/kg; p.o.) and 30 min later underwent the capsaicin test. In this test, the animals received an injection of 20 μL capsaicin (1.6 μg/paw; i.pl.) in the left hind paw, while the right hind paw received a saline vehicle (0.9% saline) injection. The animals were individually observed for 10 min following capsaicin injection, and the duration of paw licking was recorded as an indicator of nociception [7].

### 4.10. Statistical Analysis

Statistical analysis was conducted using GraphPad Prism 7.0 software (San Diego, CA, USA). For assessing the mechanisms of action of MTDZ, a one-way ANOVA was employed. To explore the therapeutic potential of MTDZ against PTX−induced peripheral neuropathy in behavioral and biochemical assays, a two-way ANOVA was utilized. Post hoc analysis was performed using Tukey’s multiple comparisons test. The data are presented as mean ± standard error of the mean (S.E.M.). Main effects are reported only if the higher-order second interaction was not statistically significant. Statistical significance was defined as *p* < 0.05.

## 5. Conclusions

In view of the data demonstrated here, MTDZ presents itself as an innovative compound capable of presenting therapeutic actions in acute nociception and neuropathic pain caused by PTX chemotherapy. Indeed, the therapeutic action of target molecules to combat the neuropathy side effect of PTX is an emerging need for cancer patients. MTDZ was able to combat the nociception caused by PTX when assessing mechanical and thermal sensitivities in male and female mice. For the first time, mechanical hypersensitivity was observed in females exposed to PTX on day 14 of the experimental protocol, and thermal hypersensitivity in females on day 11, and this hypersensitivity in females normalized by day 14. Moreover, the ability of MTDZ to reduce NOx levels and modulate Ca^2+^−ATPase activity is a possible relevant mechanism within the framework of neuropathy caused by PTX.

At the level of acute nociception, we verified the ability of MTDZ to modulate the TRPV1, glutamatergic, nitrergic, cGMP, serotonergic, and opioidergic pathways. These results provide support and robustness to the hypothesis that MTDZ-modulated pathways are capable of alleviating acute painful situations involving both inflammation and targeted activation of nociceptors.

## Data Availability

Data is contained within the article.

## Sample Availability

Samples of the compounds are available upon request from the authors.
